# Explaining #theShoe based on the optimal color hypothesis: The role of chromaticity vs. luminance distribution in an ambiguous image

**DOI:** 10.1016/j.visres.2020.10.007

**Published:** 2021-01

**Authors:** Takuma Morimoto, Kazuho Fukuda, Keiji Uchikawa

**Affiliations:** a Department of Experimental Psychology, University of Oxford, Oxford, UK; b Department of Information Design, Kogakuin University, Tokyo, Japan; c Human Media Research Center, Kanagawa Institute of Technology, Atsugi, Japan

**Keywords:** #theShoe, Color constancy, Optimal color, Illuminant estimation

## Abstract

The image of #theShoe is a derivative image of #theDress which induces vastly different color experiences across individuals. The majority of people perceive that the shoe has grey leather with turquoise laces, but others report pink leather with white laces. We hypothesized #theShoe presents a problem of color constancy, where different people estimate different illuminants falling onto the shoe. The present study specifically aimed to understand what cues in the shoe image caused the ambiguity based on the optimal color hypothesis: our visual system knows the gamut of surface colors under various illuminants and applies the knowledge for illuminant estimation. The analysis showed that estimated illuminant chromaticity largely changes according to the assumed intensity of the illuminant. When the illuminant intensity was assumed to be low, a high color temperature was estimated. In contrast, assuming high illuminant intensity led to the estimation of low color temperature. A simulation based on a von Kries correction showed that the subtraction of estimated illuminants from the original image shifts the appearance of the shoe towards the reported states (i.e. gray-turquoise or pink-white). These results suggest that the optimal color hypothesis provides a theoretical interpretation to #theShoe phenomenon. Moreover, this luminance-dependent color-shift was observed in #theDress phenomenon, supporting the notion that the same trigger induces #theShoe.

## 1 Introduction

In February 2015 a photograph of a dress became a viral internet phenomenon; the population was divided on whether they saw the image of a dress as blue and black, or as white and gold. This phenomenon spread as #theDress and convincingly demonstrated that individuals’ color vision systems possess striking variations. One fascinating aspect of the phenomenon is that different observers experienced different color appearances whilst conventional color illusions “deceive” people in the same way. The dress image was recognized as a novel phenomenon in the vision science community and intensive efforts were made to seek plausible accounts to decode this mysterious image.

A substantial number of studies on #theDress exists to date. Some of the earliest research proposed a color constancy hypothesis to describe the phenomenon: some people assume a warm illuminant, and others assume a cool illuminant falling on the dress surface (Lafer-Sousa et al., 2015, Rogers, 2015). Many other studies shared this view, deepened theoretical arguments and accumulated empirical evidence (Brainard and Hurlbert, 2015, Wallisch, 2017, Toscani et al., 2017, Witzel et al., 2017). Thus, a major focus in past studies was to identify the factor that causes people to infer different illuminants falling onto the dress. Proposed accounts range across various stages of visual processing. For example, individual differences in pupil size (Vemuri, Bisla, Mulpuru, & Varadharajan, 2016) and macular pigment density (Rabin et al., 2016) are reported to show associations with dress appearance. At a post-receptoral level the strength of blue-yellow asymmetry was shown to correlate with the color naming (Winkler et al., 2015). The importance of the individual variations along blue-yellow axis is further supported by Feitosa-Santana et al. (2018), who explored various color tests: color naming and matching, anomaloscope matching, unique white measurement and color preference rating. One of the earliest studies took a big-data approach capitalizing upon an online survey (Lafer-Sousa, Hermann, & Conway, 2015) and suggested that age and gender seem to be related to the perception of the dress. Some studies showed that individuals’ chronotypes are weakly associated with dress percept (Lafer-Sousa and Conway, 2017, Aston and Hurlbert, 2017). Furthermore, a twin study reported the impact of genetic factor is limited, and thus environmental factors need to play a role (Mahroo et al., 2017). Neural mechanisms to underpin the dress phenomenon were also identified using fMRI (Schlaffke et al., 2015) and more recently in the electroencephalogram (Retter et al., 2020). It was found that the activation of areas that are known to be associated with top-down modulation are associated with perception of #theDress, implying the influence of high-level cognition on judging dress appearance.

Interestingly, various studies demonstrated that it was possible to decrease the ambiguity by manipulating the dress image. Dixon and Shapiro (2017) pointed out that filtering the dress image by a low- or high-pass filter removes ambiguity, suggesting how individual visual systems extracting low and high spatial frequency chromatic components might explain the difference. Similarly, it was shown that color naming changes by occluding the image (Daoudi et al., 2017), by exposing observers to a brightness illusion (Hugrass et al., 2017), or by embedding explicit cues about the illuminant (Lafer-Sousa et al., 2015, Witzel et al., 2017).

#theShoe is a later generation of #theDress, which also elicited observer-dependent color experiences. A majority of observers reported that the shoe has gray leather and turquoise lace, but some people perceived the shoe with pink leather and white laces (Werner et al., 2018). However, the shoe phenomenon has been explored very little (Daoudi et al., 2020) considering the number of studies on the dress. Consequently, it largely remains unclear whether findings about the dress image can be applied to #theShoe phenomenon.

In our previous study of the dress image (Uchikawa, Morimoto, & Matsumoto, 2017) we applied a computational model which we developed for how observers estimate the color of light illuminating a scene. In the physical world of lights and reflecting surfaces the set of observed surface colors depends on the color of the illumination. The model derives an estimate of the illuminant from this constraint which we called the “optimal color hypothesis”. In this paper we tackled #theShoe phenomenon based on the optimal color hypothesis, aiming to extract hidden image cues causing the ambiguity.

**Fig. 1:**
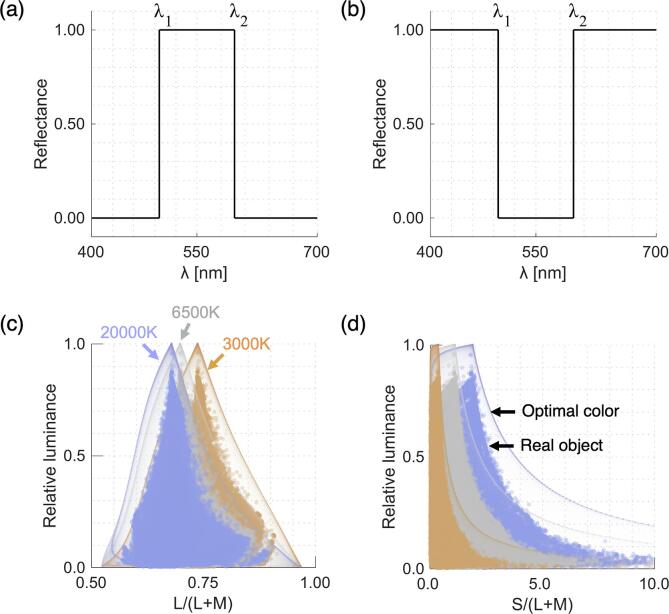
(a), (b) An example of band-pass and band-stop optimal color. (c), (d) Chromaticity versus luminance distributions of 102,721 optimal colors and 49,667 real objects under illuminants of 3000 K, 6500 K and 20000 K.

A full description of the optimal color model is available elsewhere (Morimoto et al., 2020), but here we will introduce the basic concept. An optimal color is a hypothetical surface that consists of only 0% and 100% reflectances. There are band-pass and band-stop types as shown in Fig. 1(a) and (b). If we parametrically vary λ_1_ and λ_2_ (λ_1_ < λ_2_), we can define numerous optimal colors. Panels (c) and (d) show the color distribution of 102,721 optimal colors and 49,667 real objects (SOCS, ISO/TR 16066:2003) under the illuminants of 3000 K, 6500 K and 20000 K on the black body locus. An important aspect of optimal colors is that since they have an extreme reflectance function, they have the highest luminance across any colors that have the same chromaticity. Therefore, the distribution of optimal colors visualizes a physical upper luminance boundary over chromaticities under a specific illuminant. An optimal color distribution always peaks at a full-white surface, and thus the peak of cone-like shape indicates the chromaticity and the intensity of the illuminant. Panels (c) and (d) show that the color variation of real objects seems to be rich enough to fill the large portion of optimal colour distributions. This suggests that our visual system might have access to physical upper boundary simply through seeing colors in a daily life, and thus it may be possible for us to internalize the shape of optimal color distribution under typical illuminants (e.g. blue-yellow direction). Also, it is notable that the real object colour distributions behave in approximately the same way as those of optimal colors in response to illuminant color change. In other words, in the real world there is a strong association between the illuminant color and how surface colours distribute. If the visual system is aware of such statistical regularities, it may be possible to use this constraint as a prior to estimate the illuminant influence in a scene. Based on these ideas, the optimal colour hypothesis is defined as follows: our visual system infers the illuminant influence by selecting the most plausible optimal colour distribution that best fits a given chromaticity-luminance distribution. The optimal color distribution indicates the upper limit that the luminance of a real surface can theoretically reach. Thus, in a scene that does not includes a light source or specular reflection, it is important that any color in the scene does not exceed the selected optimal color distribution.

If the optimal color hypothesis is adopted by human observers the model might be able to guide us to understand why the shoe image can be interpreted by being illuminated by different illuminants. Such an attempt revealed that estimated color temperature of illuminants largely shifted as a function of estimated illuminant intensity. When the illuminant intensity was estimated to be low, the best-fit color temperature was high. However, as assumed illuminant intensity increased the estimated color temperature accordingly decreased. Using the illuminants estimated by the model we applied von Kries correction to the original image to simulate the appearance of the shoe when the estimated illuminant influence was subtracted. The corrected images seemed to change their appearances continuously between reported states (i.e. turquoise and gray or pink and white). In summary, our model accounted for #theShoe phenomenon in a similar way that it explained #theDress phenomenon.

## 2 Analysis method

### 2.1 Analyzed image and color distribution

**Fig. 2:**
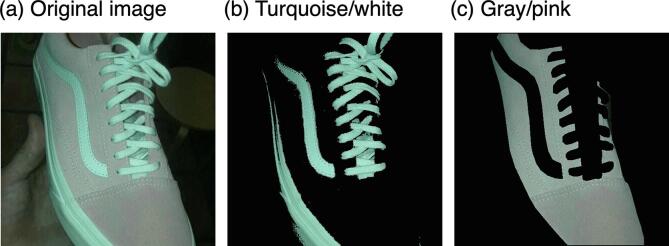
(a) The original image of #theShoe. Color appearance of the image is mainly divided into two groups: turquoise and gray or white and pink. (b), (c) Segregated regions that appear turquoise or white and gray or pink, respectively. (For interpretation of the references to color in this figure legend, the reader is referred to the web version of this article.)

Panel (a) in Fig. 2 shows the original image of the shoe. For the analysis, we first segregated the original image to (b) turquoise or white and (c) gray or pink regions. The original image stored RGB values at each pixel, but the conversion from RGB to cone response is dependent on a monitor on which the image is presented. In the analysis, we assumed that we present the image to an ordinary CRT monitor (NEC, FP2141SB, 21 in., 1600 × 1200 pixels). Using the spectral measurement of the RGB phosphor and gamma function, we converted RGB values to LMS cone responses based on Stockman and Sharpe cone fundamentals (Stockman & Sharpe, 2000). The cone responses were further converted to MacLeod-Boynton chromaticity coordinates (MacLeod & Boynton, 1979), where L/(L + M) and S/(L + M) of the equal energy white was scaled to have 0.708 and 1.000.

**Fig. 3:**
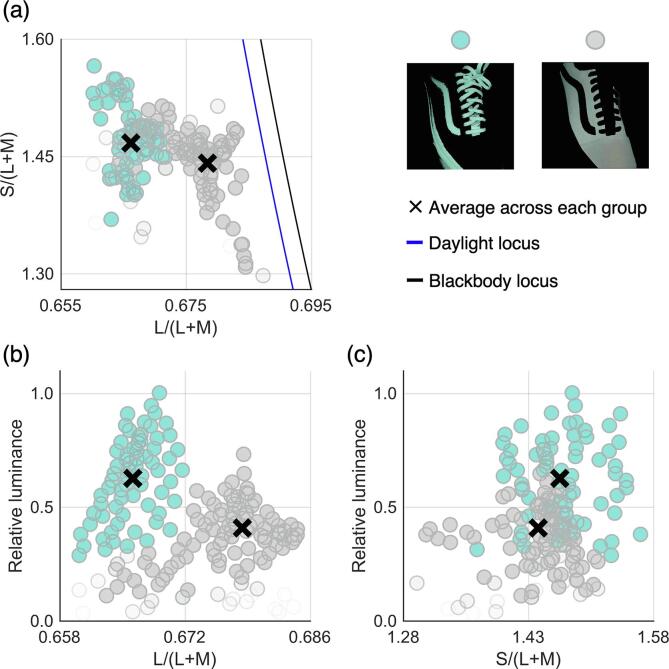
Color distribution of #theShoe image. (a) Chromatic distribution plotted on MacLeod-Boynton chromaticity diagram. (b) L/(L + M) versus luminance distribution. (c) S/(L + M) versus luminance distribution. The luminance is normalized by the maximum luminance across all pixels in the shoe image and thus labelled as relative luminance. Turquoise circles are pixels belonging to the turquoise/white region (panel (b), Fig. 2). Gray circles denote pixels in the gray/pink region (panel (c), Fig. 2). Black cross symbols indicate mean colors across each region that were used for subsequent analysis. (For interpretation of the references to color in this figure legend, the reader is referred to the web version of this article.)

Fig. 3 shows the color distribution of the shoe image. The turquoise and gray circles show the chromaticity and luminance of pixels that belong to the turquoise/white region (53,398 samples) and the gray/pink region (81,349 samples), respectively. The black cross symbols indicate mean colors across each region. The panel (a) shows that chromatic distribution of #theShoe image deviates from the blackbody locus and daylight locus. The panel (b) and (c) show the L/(L + M) vs. luminance and S/(L + M) vs. luminance distributions. Note that the absolute luminance level of #theShoe image depends largely on the monitor on which the image is presented. Thus in this study all luminance values were normalized by the maximum luminance value across all pixels in the shoe image. We used two mean colors (black cross symbols) for the subsequent analysis instead of a whole color distribution. There are two reasons for this. First, our model is susceptible to an outlier as it is assumed that any point should not exceed the optimal colour distribution. For example, if there is a single pixel that is much lighter than others, optimal colour distribution needs to cover the light surface, and consequently the fitting results might be severely biased. Second, the ambiguous image such as #theDress and #theShoe may occur due to poor chromatic information in the image (i.e. only 2 colors), and thus we decided to incorporate this curious information limit in #theShoe image into our analysis. Using mean values is not an only way, but it is a simple way to bypass these concerns. This use of mean color is also consistent with our previous analysis, allowing for compatibility of results between the present and the previous study.

### 2.2 Illuminant estimation based on the optimal color model

We applied the optimal color model to estimate the influence of illuminant on the shoe. In the model framework, it is assumed that the model stores the chromaticity and luminance of all possible optimal colors under 3,478 candidate illuminants: 37 color temperatures from 2000 K to 20000 K with 500 steps × 94 intensity levels from 0.671 to 1.25 with 0.00623 steps. The goal of the model is to find illuminants under which the optimal color distribution and observed color distribution match well, evaluated by weighted root-mean-squared-error (WRMSE). There were two analyzed colors *S_1_* and *S_2_* (namely, mean colors across the turquoise/white region and the gray/pink region, respectively), and their luminances can be written as *Ls_1_* and *Ls_2_*. If we define the luminance of the corresponding optimal colors at their chromaticities as *Lo_1_* and *Lo_2_*, *WRMSE* values for all candidate illuminants are calculated using Eq. (1).

(1) $$\mathrm{WRMSE}=\sqrt{\frac{w_{1}{\left(L_{s1}-L_{o1}\right)}^{2}+w_{2}{\left(L_{s2}-L_{o2}\right)}^{2}}{w_{1}+w_{2}}}$$

$$w_{i}=\frac{L_{\mathrm{si}}}{L_{\mathrm{oi}}}\left(i=1,2\right)$$

We put a weighting *w_i_* on the error to give a greater weighting to lighter surfaces. This is based on the past finding that higher luminance surfaces had greater influences on observers’ estimation of illuminant colour (Uchikawa, Fukuda, Kitazawa, & MacLeod, 2012). Note that *w_i_* reaches 1.0 when *Lsi* (surface luminance) perfectly matches *Loi* (optimal color luminance). We excluded any illuminants under which either (or both) of the two colors exceeds the optimal color distribution. When the illuminant intensity level was lower than 0.671, illuminants of any candidate color temperatures were excluded. This is why we used 0.671 as the lower boundary of candidate intensity level. Then, we looked for illuminants from the remaining candidates under which the value of *WRMSE* becomes small*.* If the model can find small *WRMSE* values for multiple candidate illuminants that have largely different color temperatures, it would imply that the shoe image holds the ambiguity about illuminant influence. The following section describes that this was the case.

## 3 Results

**Fig. 4:**
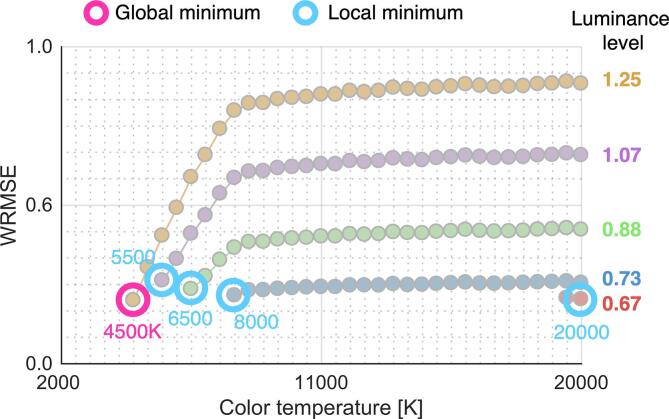
*WRMSE* plot as a function of color temperature (2000 K to 20000 K with 500 steps) at different intensity levels (0.67, 0.73, 0.88, 1.07 and 1.25). Each circle indicates the *WRMSE* value for one candidate illuminant that has a specific color temperature and an intensity level. When one or two analyzed colors exceeded the optimal color distribution of the candidate illuminant, that illuminant was excluded from the analysis. This is why some regions have no data (e.g. there is no data points below 19500 K for intensity level 0.67).

Fig. 4 shows the *WRMSE* plot as a function of the color temperature at five luminance levels. Notice that some data points are not presented (e.g. there is no data below 19500 K for luminance level 0.67). This is because those candidate illuminants were rejected as one (or two) of the analyzed colors exceeded the optimal color distribution. Additionally, these five luminance levels were selected arbitrarily, but data exist at other luminance levels.

First, the global minimum *WRMSE* value across all candidate illuminants was found at color temperature 4500 K and luminance level 1.25. However, as we decreased the luminance level low color temperature illuminants were rejected and the trajectory of *WRMSE* curve changed. As a result, the best-fit color temperatures increased from 4500 K to 5500 K, 6500 K, 8000 K and eventually 20000 K.

**Fig. 5:**
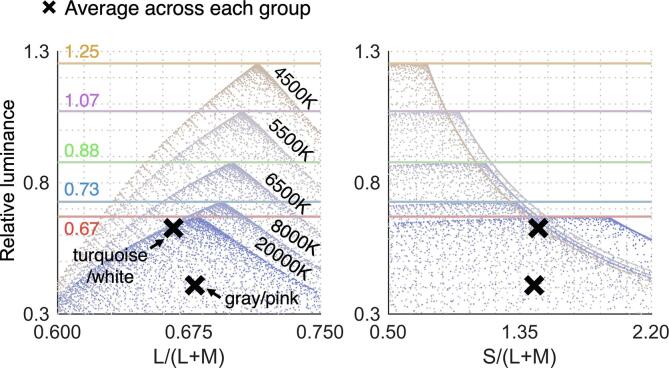
Best-fit optimal color distributions at different intensity levels. We see that estimated color temperature continuously changes from high to low color temperature as the estimated illuminant intensity increases.

Fig. 5 shows schematic illustration of how the best fit optimal color distributions change as a function of luminance level. At the luminance level 0.67 an optimal color distribution under 20000 K was found to fit the best. This is because that turquoise/white surface cannot be covered by the optimal color distribution under low color temperature illuminants when the intensity is low. However, if we increase the intensity level this excess no longer happens, and the best-fit color temperature consequently decreased.

Overall we found that depending on the luminance level of illuminants we are searching through *WRMSE* values converged to different color temperatures. It is worth noting that although we found an illuminant of 4500 K as the global minimum (the magenta circle in Fig. 4), the *WRMSE* value is nearly the same as those of local minimums (cyan circles in Fig. 4). In other words, these candidate illuminants are nearly equally plausible, which might explain why individuals put different interepretaions on the illuminant influence.

Next, using the estimated illuminants we simulated the color appearance of the shoe when those illuminant influences are discounted from the original image. Specifically we applied a von Kries correction which scales cone signals *L*, *M* and *S* at each pixel by the proportion between cone responses under equal energy white (*Lw*, *Mw*, and *Sw*) and under an estimated illuminant (*Le*, *Me*, and *Se*) to simulate cone signals as if it were placed under an equal energy white illuminant. This manipulation is written as equation (2).

(2) $$\left(\begin{array}{c} L\text{'}\\ M\text{'}\\ S\text{'} \end{array}\right)=\left(\begin{array}{ccc} \mathrm{Lw}/Le & 0 & 0\\ 0 & \mathrm{Mw}/Me & 0\\ 0 & 0 & \mathrm{Sw}/Se \end{array}\right)\left(\begin{array}{c} L\\ M\\ S \end{array}\right)$$

**Fig. 6:**
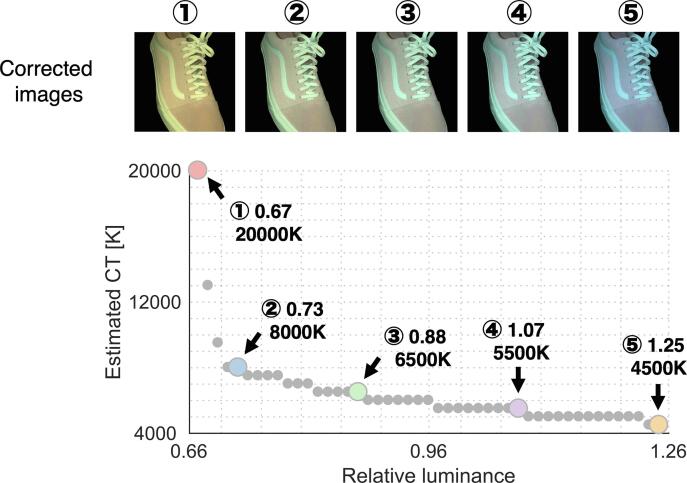
The gray small and colored circles together show the estimated color temperatures (CT) as a function of assumed at 47 luminance levels from 0.67 to 1.25 with 0.0125 steps. Five colored circles are five representatives estimated color temperatures: 20000 K, 8000 K, 6500 K, 5500 K and 4500 K. Images above show corrected images where the influence of illuminant was discounted from the original image based on von Kries scaling (detailed in the main text). Color appearance of the shoe largely changes depending on the corrected color temperatures.

Obtained *L’*, *M’*, and *S’* values were then converted to RGB values for the display presentation. Fig. 6 provides a summary of the analysis with von Kries corrected images. The gray small and colored circles together show how the best-fit color temperatures change as a function of assumed intensity (47 levels from 0.67 to 1.25 with 0.0125 steps). The five colored circles are representative data points used as examples in Fig. 4, Fig. 5. We see that estimated color temperature continuously changes as opposed to bimodally. The von Kries scaled images shown at the upper part of the figure demonstrates that the color appearance of the shoe dramatically changes depending on the color temperature of corrected illuminants. When the image is corrected by high color temperature (e.g. ①), the shoe potentially appears white and pink. In contrast, the correction by low color temperature (e.g. ⑤) seems to yield a turquoise and gray appearance. Note that the effect of this simulation depends on presented monitor and individuals.

## 4 Discussion

A major finding in the present study is that our model suggested more than one plausible illuminant. The *WRMSE* values for the global minimum and local minima were found to be fairly close, which provides a potential reason why the image is open to various interpretations about the illuminations. Estimated illuminant color temperatures changed depending on the assumed intensity of illuminants. Because the turquoise/white region has higher luminance than gray/pink region (as demonstrated in Fig. 5), the low color temperature cannot be a candidate illuminant when the illuminant intensity is assumed to be low. This observation suggests that how luminance values of surfaces are associated with their chromaticities (e.g. geometry of color distribution) plays a crucial role. However, it is important to note that since the shape of *WRMSE* function is purely determined by the combination of 2 colours, there may be an image which might “deceive” our model. For example, it is possible to manually generate objects that have the same colour distribution as #theShoe, and the ambiguity might not occur with every object. Therefore, we do not intend to claim that the shape of colour distribution alone is sufficient to produce bi-stable perception, and we believe that other factors need to play a role to induce ambiguity which is further argued in the final paragraph of this section.

It remains a curious open question as to what factors in #theShoe image determines what value individuals assign to the intensity of the illuminant. The present study does not directly answer this question, but there might be a hint in a past observation. In our previous dress study (Uchikawa, Morimoto, & Matsumoto, 2017), we conducted a white setting experiment where participants were asked to adjust the chromaticity and the luminance of a test field embedded in the dress surface to measure observers’ estimates of illuminant intensity and illuminant chromaticity. We found that blue-black perceivers tended to estimate the illuminant intensity to be high while white-gold perceivers estimated a low intensity. Importantly, we also found that this observation held nearly perfectly even for an achromatic dress image which has the same luminance distribution as that of the original dress image. This past observation allows us to speculate that the variation in illuminant intensity estimation across individuals has little to do with the chromatic component of #theShoe image and more to do with luminance cues in the image such as spatial structure of the image.

**Fig. 7:**
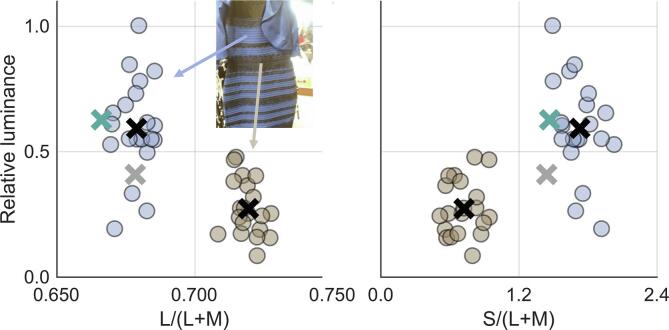
Chromaticity versus relative luminance distribution of #theDress image. Blue and brown circles are 20 pixels sampled from the blue/white and the black/gold region in the image, respectively. Black cross symbols indicate mean chromaticities across each region. Green and gray cross symbols are the mean color across turquoise/white and gray/pink regions in the shoe image for the sake of comparison. (For interpretation of the references to color in this figure legend, the reader is referred to the web version of this article.)

A similar intensity-dependent color-shift was also found in the analysis of the dress (Uchikawa, Morimoto, & Matsumoto, 2017). For comparison, Fig. 7 shows a chromaticity versus luminance distribution of the dress image, formed by 20 pixels sampled from each of the blue/white and black/gold regions. Fig. 3, Fig. 7 allow us to see that the geometry of chromaticity versus luminance distributions for the dress and shoe image are somewhat similar, although the range of chromaticity seems to be much wider for the dress. This similarity in the relative shape of color distributions seems to underlie ambiguities in both images.

Early studies made a notable observation that chromaticity distribution of #theDress image spread tightly along the daylight locus (or blue-yellow direction more generally), which might cause difficulty in judging whether chromatic variation over the dress surface stems from a surface color change or an illuminant color change (e.g. Lafer-Sousa et al., 2015, Gegenfurtner et al., 2015, Winkler et al., 2015). However, this observation curiously does not seem to hold for #theShoe image as shown in the panel (a) in Fig. 3. The whole distribution deviates from the daylight locus and the direction along which chromaticities distribute is not well aligned with the daylight locus. Our model shows that it is the shape of chromaticity vs. luminance distribution rather than the chromaticity distribution alone that might introduce the ambiguity about the illuminant falling onto the shoe surface. This might explain why bi-stable percept occurs for #theShoe image despite the violation of chromaticities clustering around the daylight locus.

In the present study, we restricted our search of candidate illuminants to black-body locus. We believe that such analysis demonstrated that if humans do have priors along the blue-yellow direction, we can potentially explain why the shoe image causes the bi-stable perception. However, as the chromatic distribution of #theShoe image is away from the locus as shown in panel (a) of Fig. 3, candidate illuminants outside the black-body locus might yield a better fitting of the model. We restricted candidate illuminants to the blue-yellow direction mainly because our model relies on prior knowledge about the illuminants (i.e. optimal colour distribution), and we assume that such prior should be learned through the observation of many surface colours under various illuminants in a daily life. Therefore, it seemed slightly unnatural to assume that our visual system knows the theoretical upper boundary under atypical illuminants outside the blue-yellow axis. A further complication is that calculating chromaticity and luminance of optimal colours under a specific illuminant requires defining the spectral power distribution of the illuminant. However, outside the black-body locus or daylight locus, we do not have strong evidence as to what sort of spectral shape we should assume (or our visual system assumes). It will be interesting to examine what illuminant prior our human visual system holds and to expand the optimal color model to a wider region of color space based on the empirical evidence.

Fig. 6 suggests that best-fit color temperature changes *continuously* as a function of assumed intensity as opposed to *discretely*. In other words, it is possible the color appearance of the shoe image might also vary gradually from one individual to another, which seems to be demonstrated by a set of von Kries corrected images in Fig. 6. This casts doubt on the notion that #theShoe and #theDress are a bi-modal phenomenon. Regarding #theShoe phenomenon, Werner et al. (2018) indeed showed that observers were divided into three groups: gray-turquoise (53%), pink-white (34%) and pink-turquoise (11%). Furthermore, it is worth pointing out that the color appearance of #theShoe and #theDress was reported by categorical color naming which express the colour discretely rather than continuously. Thus, we speculate that the colour appearance of the shoe might indeed vary in a continuous fashion, and it is the categorical colour naming that make the phenomenon appear bi-modal. This idea seems to be supported by a past finding by Gegenfurtner et al. (2015) in which participants were asked to select the colour chip that represents the colour of the dress and found that selected colour chips spread widely in color space rather than bi-modally. Also in our previous study (Uchikawa, Morimoto, & Matsumoto, 2017), we found that observer’s white point settings of a test patch embedded in the dress surface did spread continuously along the daylight locus.

One question raised from the shoe and the dress images is whether such ambiguous images happen because the object has only two color categories. It is worth reminding ourselves that regardless of whether the image is the shoe or the dress, color constancy always imposes a challenge of ambiguity about surface and illuminant colors to our visual system. In an extreme scene where only one surface exists, color constancy is essentially lost. In this sense the success of color constancy heavily depends on the number of surface colors available in a scene. Many influential color constancy algorithms such as mean chromaticity (Buchsbaum, 1980) or chromaticity-luminance correlation (Golz & MacLeod, 2002) requires a sufficient number of surfaces. Our optimal color model is not an exception. As more surface colors become available in a scene, the shape of color distribution becomes clearer, leading to better and unique model fitting. It is worth emphasizing that the basis of the optimal color model is that if the chromaticity versus luminance distribution of a given scene behaves in a similar way as those of optimal colors, the visual system can effectively estimate the illuminant color. It is probably not the case for the shoe image (and the dress image), which presumably provides the main reason why our model estimated more than one candidate illuminant in the analysis.

Recent papers by Wallisch and Karlovich, 2019, Witzel and Toscani, 2020 proposed a way to generate an ambiguous image. Importantly it was shown that the ambiguity still remains when the chromatic property of the dress image was mapped onto a different bicolored object. This result supports the importance of color distribution, which is consistent with the finding in the present study. Also, we agree with the view that generating ambiguous images freely is a powerful way to show that we understand why ambiguity happens. Based on the analysis in this study we would suspect that the following conditions seem to be key to generating a bi-stable image. Firstly, a scene needs to have a color distribution such that it does not agree well with the optimal color distribution and the best-fit color temperature (preferably largely) changes depending on assumed intensity level. Second, by correcting the influence of estimated illuminants from the image the chromatic coordinates must cross the border of color categories so that people use a different color name. Fig. S1 in supplementary material shows how chromaticities change in response to von Kries correction. Thirdly, the image needs to pose an ambiguity about illuminant intensity. This would be important because if the intensity of the illuminant is obvious, we may not need to search candidate illuminants over various intensity levels. However, it is an open question as to whether these are merely necessary conditions or sufficient conditions. For example, the spatial structure was shown to be important in #theDress phenomenon (Hesslinger and Carbon, 2016, Jonauskaite et al., 2018). Taking these points together, we believe that #theShoe and #theDress phenomena are complex and a single model does not provide a comprehensive understanding. It is therefore important that studies take a wide range of approaches to tackle the problem and accumulate evidence to provide a comprehensive perspective. In any case, one advantage to having a computational model is that we can theoretically test whether a newly generated image is likely to induce a bi-stable percept. We believe that extending this study in this direction will help further our understanding of the nature of these curious bi-stable images.

## CRediT authorship contribution statement

**Takuma Morimoto:** Conceptualization, Methodology, Software, Validation, Formal analysis, Investigation, Resources, Data curation, Writing - original draft, Writing - review & editing, Visualization, Project administration, Funding acquisition. **Kazuho Fukuda:** Conceptualization, Methodology, Writing - review & editing, Supervision, Project administration, Funding acquisition. **Keiji Uchikawa:** Conceptualization, Methodology, Writing - review & editing, Supervision, Project administration, Funding acquisition.
