# Supplementary material for: Explaining #theShoe based on the optimal color hypothesis: The role of chromaticity vs. luminance distribution in an ambiguous image
Source: Vision Res. 2021 Jan;178:117–23. doi: 10.1016/j.visres.2020.10.007 (PMC7116573; doi:10.1016/j.visres.2020.10.007)
Supplement: Supplementary data 1 [file mmc1.docx]

**Supplementary material**

**The effect of von Kries correction on mean chromaticity**

Figure S1 shows how the mean chromaticities of the lace part and leather part change in response to a von Kries correction. Note that this figure shows a result of subtracting an illuminant color, which thus indicate illuminant-free reflectance-based representation of chromaticities. The black circles and the black triangle symbols denote the mean color across lace part and leather part of the original image, respectively. We see that when we correct the original image by low color temperature (as in the case of ⑤), the mean color shifts towards low L/(L+M) and high S/(L+M). In contrast, when the image is corrected by high color temperature (as in the case of ①) colors shift towards the direction of high L/(L+M) and low S/(L+M). We suspect that as a result of these transformations chromatic coordinates cross color categories, which consequently induces observer-dependent color naming.


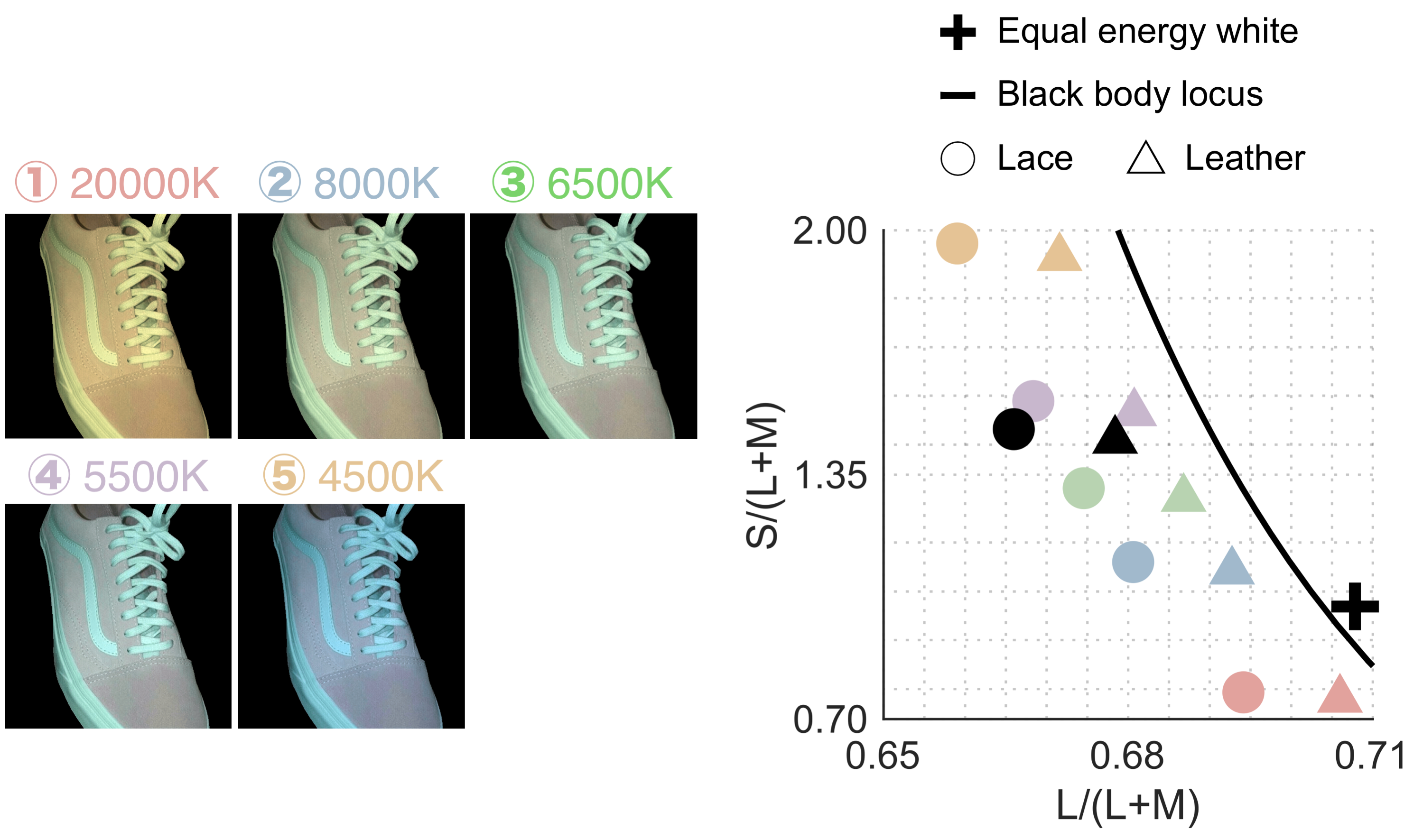


Figure S1: How chromaticities of the lace (circle symbols) and leather part (triangle symbols) changes in response to a von Kries correction. These chromaticities correspond to reflectance-based representation which is free from illuminant influence. Color label indicates the corrected color temperature as shown at the left part of the figure (corrected by ①: 20000K, ②: 8000K, ③: 6500K, ④: 5500K, and ⑤: 4500K). The black circle and black triangle symbols indicate the chromatic coordinates of original image. Note that the color label is kept the same as Figure 6.
